# Simultaneous allocation of buffer capacities and service times in unreliable production lines

**DOI:** 10.1080/00207543.2023.2168310

**Published:** 2023-01-24

**Authors:** Khelil Kassoul, Naoufel Cheikhrouhou, Nicolas Zufferey

**Affiliations:** a Geneva School of Economics and Management, GSEM – University of Geneva, Geneva, Switzerland; b Geneva School of Business Administration, University of Applied SciencesWestern Switzerland, HES-SO, Geneva, Switzerland; c IFM Business School, Geneva, Switzerland

**Keywords:** Buffer and service time allocation, finite perturbation analysis, genetic algorithm, production lines, production rate

## Abstract

Simultaneous allocation of service times and buffer capacities in manufacturing systems in a random environment is a NP-hard combinatorial optimisation problem. This paper presents a sophisticated simulation-based optimisation approach for the design of unreliable production lines to maximise the production rate. The proposed method allows for a global search using a Genetic Algorithm (GA), which is coupled with Finite Perturbation Analysis (FPA) as a local search technique. Traditional techniques based on perturbation analysis optimise decision variables of the same nature (e.g. service time only, buffer capacity only), whereas the proposed technique simultaneously provides an allocation of service times and buffer capacities. One of the main focuses of this paper is the investigation of the persistence or absence of the buffer and service rate allocation patterns which are among the most essential insights that come from designing production lines. The results show the superiority of the combined GA-FPA approach regarding GA and FPA in terms of solution quality and convergence behaviour. Moreover, considering instances ranging from 3 to 100 machines, our numerical experiments are in line with the literature for small instances (as similar allocation patterns are identified in our work), but important differences are highlighted for medium/large instances.

## 1. Introduction

Serial production lines are widely employed for mass and batch production systems. These lines are composed of machines connected in series with buffers between each pair of adjacent machines. Items/units (products, components, or parts) move through these machines according to predefined sequences. In such production lines, material flow disruptions can be caused by different factors such as service time variations, machine repair cycles, machine failures, or specific events linked to buffer levels such as starvation or blocking. When these types of disruptions occur, the performance of the production lines is impaired, and their efficiency is reduced since even a small change in the design parameters may lead to significant performance losses. Therefore, allocating optimal buffer capacities and the assignment of appropriate service time to each machine is fundamental to enhance the production rate (PR) and to reduce investment costs (either in the form of work-in-process inventory costs, floor space use or capital investment). In addition, considering a joint Buffer and Service Time allocation Problem (BSTAP) is a challenging problem. The computational complexity of this problem is NP-Hard, as considering the Buffer Allocation Problem (BAP) is in itself NP-Hard (Smith and Cruz 2005; Dolgui et al. 2013).

Several studies have been conducted on production-lines design and optimisation. Much of the research has focused on the BAP. It has been a central theme that has attracted attention over the last 30 years (Papadopoulos, Li, and O’Kelly 2019). Demir, Tunali, and Eliiyi (2014) and Weiss, Schwarz, and Stolletz (2019) have reviewed in detail the literature on BAPs. Despite the substantial studies dedicated to the design of such systems, only a few publications deal with the BSTAP.

Furthermore, various methods that are related to the design of manufacturing systems are based on coupling simulation to optimisation techniques. These methods require a high number of iterations or replications and a huge amount of time, which makes their implementation in industry difficult (Shi and Men 2003). To reduce the time required to reach convergence, this paper uses Perturbation-Analysis (PA) methods that can reach good solutions on the basis of a single simulation run of the model, which reduces the convergence time (Ho and Cao 2012).

Considering the simultaneous allocation of buffer capacities and service times, a contribution of this paper is to combine the Finite Perturbation Analysis (FPA) and the Genetic Algorithm (GA). Indeed, such methods have been proved to be efficient in the case of BAP (Kassoul, Cheikhrouhou, and Zufferey 2022). In line with other techniques having a learning mechanism (Schindl and Zufferey 2015; Thevenin and Zufferey 2019), GA has a good exploration ability as it can quickly generate a variety of solutions that are spread over a large portion of the solution space. In contrast, FPA has a good exploitation ability as it can intensify the search around some promising solutions by determining an exploration direction in the search space provided by the gradient of the PR with respect to the system's parameters. Another goal of this paper is to develop (near-)optimal solutions for the BSTAP in unreliable production lines for various configurations and different system sizes. The impact of the topology of the (near-)optimal buffer-capacities and service-time allocations is one of the main goals of this study. Indeed, it helps the involved decision maker in designing the production line. To the best of our knowledge, the considered problem differs from all problems in previous works since the very scarce literature on the BSTAP deals only with reliable machines.

The remainder of the paper is organised as follows. Section 2 gives a literature review on the BSTAP. Section 3 formulates the problem in terms of a mathematical programming model and presents the detailed approach for solving the simultaneous allocation problem of both buffer capacities and service times. The development of the optimisation technique and related assumptions are presented in Section 4. In Section 5, various experiments are presented. Finally, Section 6 provides conclusions and future research directions.

## 2. Literature review

There are three main allocation problems: (1) the buffer allocation problem (i.e. the identification of the buffer capacities and their location in a production or manufacturing line), (2) the service time (workload) allocation problem (i.e. determine the appropriate workload allocated to each machine), and (3) the server allocation problem (i.e. the number of machines allocated to each workstation). The literature shows that the combinatorial complexity of each of the three allocation problems is NP-hard (Xi et al. 2022). Furthermore, as the size of the problem increases, the computational complexity of the problem increases considerably. The design of production lines may involve one, two or all three of the decision variables (i.e. buffer, workload, and server). When two decision variables are concerned (i.e. the simultaneous allocation of buffers/service times, buffers/servers, or service times/servers), the context is known as a double optimisation problem. If the problem includes the simultaneous allocation of buffers, service times and severs, it is known as a triple optimisation problem. For more details on the concept of double and triple optimisation in discrete part production lines, the reader can refer to Papadopoulos et al. (2009). This section considers only the simultaneous buffer and service time allocation problem, giving the fact that the literature addressing BAP and service time allocation solely is rich and will not be discussed in this paper. Buzacott and Shanthikumar (1993) propose the first study on the simultaneous allocation of buffers and service rates. They develop an analytical method for the joint optimisation problem and note that the maximum PR is generally obtained for a balanced buffer and service time allocations (i.e. a uniform-as-possible buffer and service time allocations). Hillier and So (1995) consider small tandem queueing systems with a fixed total number of buffer spaces to maximise the throughput. They formulate the problem as a continuous-time Markov chain and show that uniform-as-possible allocations are interesting solutions, as those reported by Buzacott and Shanthikumar (1993), when the total storage space to be allocated is a multiple of the number of buffers, with less buffer spaces allocated to the end buffers rather to the centre buffers of the system. Spinellis, Papadopoulos, and Smith (2000) present an evaluative procedure for optimising small and large production line configurations using simulated annealing. They propose an approach for simultaneously balancing servers, buffers, and service times allocation on large reliable production lines. For the allocation of buffers, they obtain similar results as Hillier and So (1995), i.e. there is an accumulation of buffers to the ends of the line. However, the allocation of service times does not show the uniform-as-possible allocation and follows a decreasing rate across the line. Zhang et al. (2017) propose a simulation cutting method based on the decomposition technique to solve the discrete-event optimisation model of the joint buffer, service time, and server allocation problem. However, the computational time to solve the problem increases significantly when many iterations are needed to reach optimal allocations. Hillier and Hillier (2006) use a cost model that considers both cost per buffer space and revenue per unit of throughput to optimise simultaneously buffer spaces and service times for small lines (up to four machines). The problem is formulated as a Markov chain using both Erlang and exponential processing times. They conclude that the service times of the solutions consistently satisfy a bowl allocation (i.e. the value of service times of the first and last machine is considerably larger than the other machines) when a small number of buffer spaces is used, and that the allocation is symmetric at the interior of the line. All these works conclude that, for both storage and service times, the bowl phenomenon occurs when the number of buffer spaces is large. Cruz et al. (2012) combine a generalised expansion method with a multi-objective GA for the allocation of service times and buffers by generating solution-curves for several single-objective functions. The interest of their study is not essentially in the designs of allocation. These obtained solution curves show a compromise between total service time allocation, overall buffer allocation and throughput. Ng, Shaaban, and Bernedixen (2017) propose a multi-objective optimisation approach for unpaced production lines. They analyse four performance measures of production systems, a set of optimal patterns of workload, and buffer allocation (average buffer level, average idle times, work in process, and throughput). Their main objective is to propose a methodology for the BSTAP to real complex production lines. They find that some interesting relationships among the performance measures studied are observed when a multi-objective design framework is considered. Instead of using simulation for the evaluation of the performance of production lines, decomposition methods have been used; Diamantidis et al. (2020) use an efficient decomposition algorithm for evaluating the PR in a production line with parallel machines. The decomposition equations are derived and applied for each parallel machine at each workstation instead of substituting an equivalent machine for the non-identical parallel machines.

Spieckermann et al. (2000) propose an approach based on a GA and simulated annealing integrated with a simulation model for solving a practical buffer planning problem for a car body assembly shop. Their objective function includes buffer sizes, deviations of service times from their respective upper bounds, and the variance of service times. Optimisation aims to minimise the objective function by calibrating weights for the overall buffer space used and service times of each station. The proposed approach is evaluated using a real-life case study of a car manufacturer. They conclude, in agreement with the planning engineers of the car, that the simulation-based optimisation is a helpful tool to enhance the design process. Tempelmeier (2003) uses a decomposition method to determine the performance evaluation and optimal buffer allocation for a real-life car body assembly shop where both variable and deterministic processing time are considered. The author sets a desired throughput (obtained before the optimisation by an initial buffer allocation) and minimises the total buffer space. Then, he fixes the value of the total buffer found to maximise the throughput (target). Finally, he reduces the service time of each station until reaching the target throughput found earlier.

Cruz, Duarte, and Souza (2018) optimise the performance of general finite single-server queueing networks as well as studying simultaneously the minimisation of service times and buffer spaces with the objective of maximising the PR. They employ a GA for generating solutions of total buffer space and service times. To improve the throughput of the system, they redistribute the total buffer while keeping the optimal service-time allocations found by reorganising the buffers to be allocated along the line using simulated annealing. To evaluate their methodology, they consider the automotive assembly system proposed by Spieckermann et al. (2000). The authors show that improvements in throughput are achieved using the evolutionary algorithm under various scenarios. Recently, to solve the joint BSTAP for different open queueing network topologies in reliable lines, Smith (2018) uses a sequential quadratic-programming approach and examines the allocation patterns for small merge and split topologies (two or three stages with up seven machines). The main objective is to investigate the absence or persistence of allocation schemes of the buffers and service rates. The obtained allocation patterns corroborate, in one sense, those found in Hillier and Hillier (2006).

The literature shows a clear gap in the case of unreliable production systems. The very few studies available for the BSTAP have only focused on reliable machines. This article offers a powerful optimisation approach that significantly improves upon the state of the art. Therefore, the interest of our work is twofold. On the one hand, the development of a design technique for stochastic production systems with unreliable machines based on the simultaneous design of buffer capacities and service times will enhance the research in production-line design. On the other hand, the development of a comprehensive optimisation technique using a single-simulation run will reduce the time needed for convergence, which contributes to the development of new generation of global-local optimisation techniques.

## 3. Problem formulation and solution approach

### 3.1. Problem formulation

**Figure 1.**
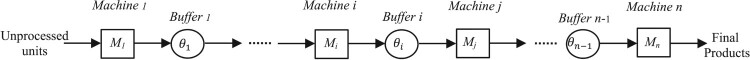
Open serial production line.

We consider open serial production lines (also denoted as flow lines, tandem lines, transfer lines) as presented in Figure 1, composed of *n* machines $\left(M_{1},\ldots ,M_{n}\right)$ connected in series and separated by (*n*-1) buffers $\left(\theta_{1},\ldots ,\theta_{n-1}\right)$, where $\theta_{i}$ denotes the buffer size located between two consecutive machines $M_{i}$ and $M_{j}$. Parts enter the system from the machine $M_{1}$, then move to the first buffer $\theta_{1}$, then to machine $M_{2}$ and so on until they reach the last machine $M_{n}$ and leave the system.

The assumptions of the system are the following.

- The first machine is never starved, and the last machine is never blocked.
- Any machine is subject to breakdown but can only fail when it is up, neither starved nor blocked. The repair and failure rates of the machines are geometrically distributed.
- The transfer times of parts from machines to stocks (and inversely) are negligible.

As machines are unreliable, the state of each machine at time *t* can be either Down (DN) if it cannot execute any operation due to internal failure, or up (UP) if it may execute tasks/parts. We define then the failure $p_{i}\;$ and repair $r_{i}\;$ probabilities of the machine *i* as follows:

-
$p_{i}=$ Probability (machine *i* is DN for next part | *i* is UP for current part)
-
$r_{i}=$ Probability (machine *i* is UP for next part | *i* is DN for current part)

The Mean Time Between Failures (resp. the Mean Time To Repair) of *M_i_* is $\text{MTB}{\text{F}}_{i}=t_{i}/p_{i}$ (resp. $\text{MTT}{\text{R}}_{i}=t_{i}/r_{i}$). When a machine is UP, it can be blocked or Full Output (FO) (resp. starved or Null Input (NI)) if its downstream (resp. upstream) buffer is full (resp. empty).

Consider the vector of decision variables $\theta =(\theta_{1},\ldots ,\theta_{2n-1})$ that has a dimension of (2*n*-1), where $\{\theta_{1},\ldots ,\theta_{n-1}\}\subset \;N$ represent the buffer capacities of available physical locations, and $\{\theta_{n},\ldots ,\theta_{2n-1}\}\subset \;R_{+}$ represent the service times of machines. Moreover, $B_{\text{max}}$ is a fixed nonnegative integer representing the total buffer space, $T_{\text{max}}$ is the total time for the manufacturing of the product, and f(θ) is the mathematical function representing the Production Rate (PR) of the line.

The design problem addressed in this paper is to allocate $B_{\text{max}}$ over (*n*-1) buffers and $T_{\text{max}}$ over *n* machines. The objective is to maximise the average PR of the production line. As in Donohue and Spearman (1993) and in Suri and Leung (1989), we consider the assumption that the total time $T_{\text{max}}$ can be distributed in some way throughout all the machines. The problem can be stated, in mathematical terms, as follows:

(1) $$\begin{array}{rl} & \text{Find}:\theta =(\theta_{1},\theta_{2}\text{,}\ldots \text{,}\theta_{2n-1})\;\text{to}\;\text{maximize}\;f(\theta ) \end{array}$$

(2) $$\begin{array}{rl} & \text{Subject}\;\text{to}:\overset{n-1}{\underset{i=1}{\sum }}\theta_{i}=B_{max};\theta_{i}\geq 0;\\ & \;\text{and}\;\text{integer}\;\text{for}\;i=1,2,\ldots ,n-1 \end{array}$$

(3) $$\begin{array}{rl} & \overset{2n-1}{\underset{i=n}{\sum }}\theta_{i}=T_{max};\theta_{i}\geq 0\;\text{for}\;i=n,\ldots ,2n-1 \end{array}$$

### 3.2. Solution approach

**Figure 2.**
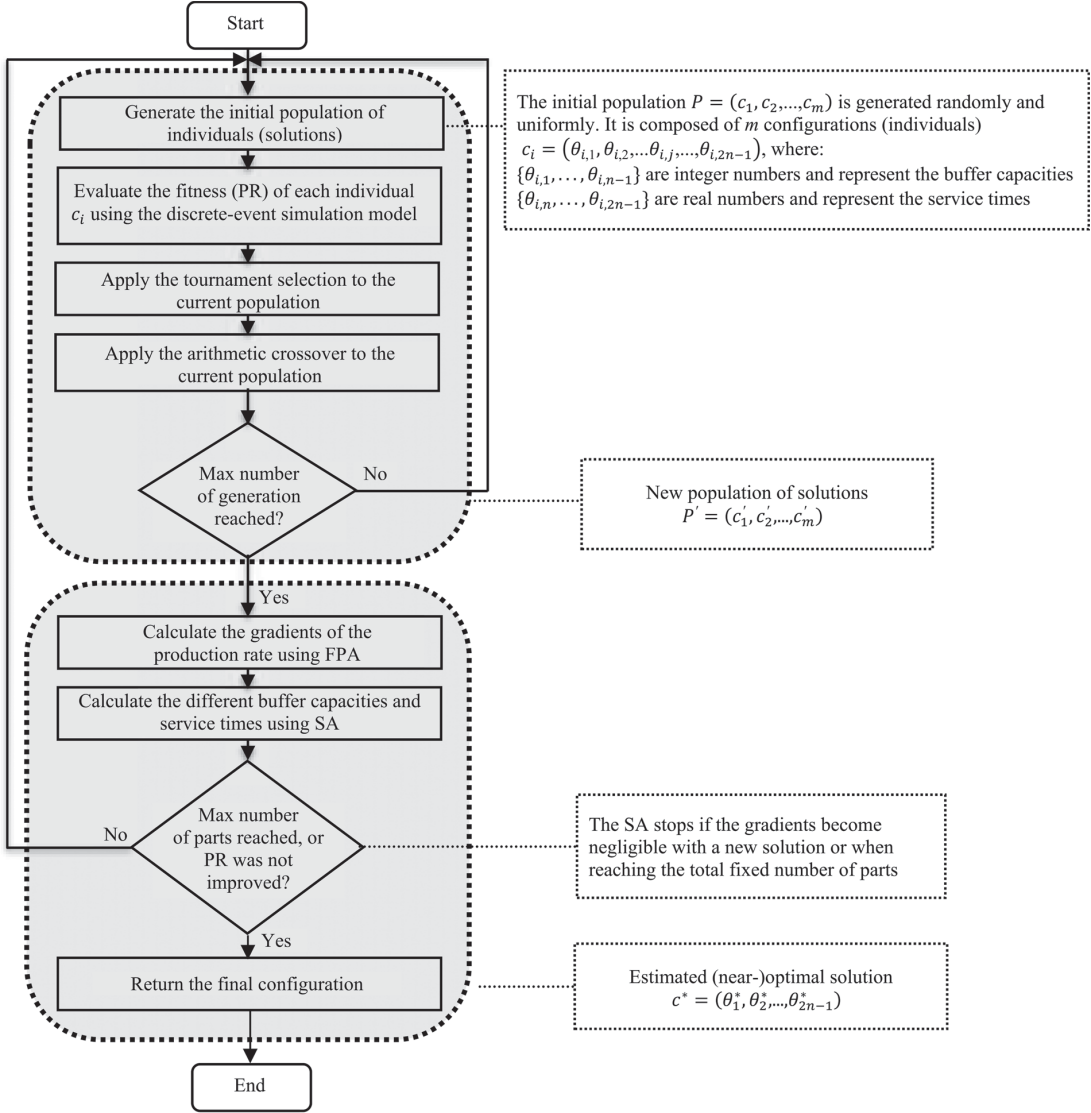
Global approach of the adopted design.

The proposed approach is a global-search procedure using a genetic algorithm (GA), which is coupled with a Finite Perturbation Analysis (FPA) local search. The goal of combining GA and FPA is to benefit from the advantages of both methods. GA allows for approaching optimal solutions in a small computing time whereas FPA can improve the solutions with the Stochastic Algorithm (SA) using the same simulation for estimating the gradients (i.e. the simulation and optimisation are conducted simultaneously). First, the GA operators are applied until a stopping condition is satisfied. Next, FPA iteratively replaces a current solution (found by GA) by a new one, until some stopping criterion is achieved. The detailed optimisation approach is presented in Figure 2, where the initial population, $P=(c_{1},c_{2}\text{,}\ldots \text{,}c_{m})$, is composed of *m* different configurations (solutions), where each solution $c_{i}=(\theta_{i\text{,1}},\theta_{i\text{,2}}\text{,}\ldots \theta_{i,j}\text{,}\ldots \text{,}\theta_{i,2n-1})$ is generated uniformly and randomly (see Kassoul, Cheikhrouhou, and Zufferey (2022) for more details on the way to generate such a population of random solutions). $\theta_{i,j}$ represents the *j*th design parameter of the *i*th configuration, where $\theta_{i,j}$ are integer (resp. real) numbers and represent the buffer capacities (resp. the service times) for j=1,2, …, n−1 (resp. j=n, …,2n−1). To approach a (near–)optimal solution $c^{*}=(\theta_{1}^{*},\theta_{2}^{*}\text{,}\ldots \text{,}\theta_{2n-1}^{*})$, the new population $P^{\prime }=(c_{1}^{\prime },c_{2}^{\prime }\text{,}\ldots \text{,}c_{m}^{\prime })$ is generated from the population *P* by applying the usual GA operators (i.e. selection, crossover, and mutation) for a fixed-number of generations. In this region, the employed stochastic algorithm (SA) relies on the gradient descent technique (Robbins and Monro 1951). SA takes the configurations of *P’* and calculates the PR gradients $\partial f(\theta )/\partial \theta_{i}$ according to the design decisions (buffer capacities and service times) $\theta_{i}(i=1,\ldots ,2n-1)$.

Consider the original discrete-event simulation of *L* parts. $\theta_{i}^{k}$ represents the decision variable $\theta_{i}$ at the iteration k. The iteration k+1 allows the progression of the variables $\theta_{i}^{k}$ by simulating p parts ( p<L) to determine a new evaluation of the gradients using FPA. The new search direction for the design solution is determined by this gradients’ evaluation. For each iteration *k*, the projections of the gradients’ vector on the following hyperplanes $\overset{n-1}{\underset{i=1}{\sum }}\theta_{i}=B_{\text{max}}$ and $\overset{2n-1}{\underset{i=n}{\sum }}\theta_{i}=T_{\text{max}}$ is estimated. In fact, for a fixed parameter $\theta_{i}$, the projected gradients allow for determining the best direction for enhancing the current solution. As the search for solutions takes place in two spaces, two iterative variants of SA are constructed.

Hence:

(4) $$\theta_{i}^{k+1}=\theta_{i}^{k}+a_{k}\left(\partial f/\partial \theta_{i}-\frac{1}{n-1}\overset{n-1}{\underset{i=1}{\sum }}\partial f/\partial \theta_{i}\right)$$

and

(5) $$\theta_{i}^{k+1}=\theta_{i}^{k}+b_{k}\left(\partial f/\partial \theta_{i}-\frac{1}{n}\overset{2n-1}{\underset{i=n}{\sum }}\partial f/\partial \theta_{i}\right)$$

where

• $a_{k}$ and $b_{k}$ are numeric suites that verify the following conditions of convergence:
(6) $$\begin{array}{rl} & \underset{k\to \infty }{lim}a_{k}=\underset{k\to \infty }{lim}b_{k}=0,\overset{\infty }{\underset{k=1}{\sum }}a_{k}=\overset{\infty }{\underset{k=1}{\sum }}b_{k}=\infty ,\\ & \overset{\infty }{\underset{k=1}{\sum }}{a_{k}}^{2}<\infty ,\;\text{and}\;\overset{\infty }{\underset{k=1}{\sum }}{b_{k}}^{2}<\infty \end{array}$$
• the term $\left(\partial f/\partial \theta_{i}-\frac{1}{n-1}\overset{n-1}{\underset{i=1}{\sum }}\partial f/\partial \theta_{i}\right)$ $(\text{resp}.\;\partial f/\partial \theta_{i}-\frac{1}{n}\overset{2n-1}{\underset{i=n}{\sum }}\partial f/\partial \theta_{i})$ in the Equation (4) (resp. the Equation (5)) represents the projection of the gradient $\partial f/\partial \theta_{i}$ on the hyper plane constraints $\overset{n-1}{\underset{i=1}{\sum }}\theta_{i}=B_{\text{max}}$ (resp. $\overset{2n-1}{\underset{i=n}{\sum }}\theta_{i}=T_{\text{max}}$).

SA stops when there is no improvement of PR with a new solution or when the total fixed number of parts is reached.

## 4. Development of the optimisation technique

### 4.1. Genetic algorithm (GA)

#### 4.1.1. Background

GA is one of the commonly used population-based stochastic metaheuristics (Chaudhry and Luo 2005). GA needs an initial population *P* of solutions to start with (such solutions are usually generated randomly to have a sufficient diversity of characteristics). Then, for a fixed number of generations, GA repeats the following steps. (1) *Selection*: some solutions of *P* are selected according to their fitness (i.e. objective-function values). (2) *Crossover*: the selected solutions are used to generate a new population $P^{\prime }$. (3) *Mutation*: the solutions of $P^{\prime }$ are randomly modified (individually).

#### 4.1.2. Selection operator

In this study, we use a robust and effective selection mechanism commonly used by GAs, which is tournament selection (Lei, Zheng, and Guo 2017). First, two individuals of the population are selected randomly. Next, their PR (obtained by the execution of the simulation model) are compared, and finally, the winner is selected for the next generation.

#### 4.1.3. Crossover operator

The arithmetic crossover operator with a constraint criterion is used (Duman 2018). First, two parents from the actual population, say $c_{1}$ and $c_{2}$, are selected. Then, the two parents are linearly combined to generate two new children $c_{1}^{\prime }$ and $c_{2}^{\prime }$ using Equations (7) and (8), where α is a coefficient selected randomly and uniformly in the interval [0, 1]. Note that an adjustment procedure is performed to fulfil constraints (2) (see our comments below).

(7) $$\begin{array}{rl} c_{1}^{\prime } & =\alpha \cdot p_{1}+(1-\alpha )\cdot c_{2} \end{array}$$

(8) $$\begin{array}{rl} c_{2}^{\prime } & =(1-\alpha )\cdot p_{1}+\alpha \cdot c_{2} \end{array}$$

#### 4.1.4. Mutation operator

Since the buffer size is an integer variable, some values of the children are decreased or increased (modified) to ensure that the value of each buffer size is an integer. The adjustment procedure and the rounding mechanism for the arithmetic crossover used in this work correspond to the mutation operator (Türkyılmaz and Bulkan 2015).

Algorithm 1 summarises the steps of the proposed GA for the considered problem.

**Table:** 

**Algorithm 1:** Genetic Algorithm (GA)
**Generate** randomly and uniformly an initial population *P* of (2*n*-1) individuals.
**Calculate** the PR of each individual using the simulation model and set $P^{\prime }=\{\}$.
**While** the new population $P^{\prime }$ does not contain (2n-1) individuals, **do**
** Repeat** n times the following procedure:
** Select** two parents randomly.
** Apply** the tournament selection according to their PR values.
** Clone** the best-selected individual (except the last one).
**Cross** two parents $c_{1}$ and $c_{2}$ using the arithmetic crossover operator
**While** constraint (2) is not satisfied, **apply** the adjustment procedure and the rounding mechanism.
**Insert** the so-obtained children solutions $c_{1}^{\prime }$ and $c_{2}^{\prime }$ in the new population $P^{\prime }$.

### 4.2. Finite perturbation analysis (FPA)

We give here an overview of the generation and propagation of perturbation through FPA. A more comprehensive work on the generation and propagation rules is presented in (Cheikhrouhou 2001). The basic idea of the Perturbation Analysis (PA) technique is to analyse a nominal sample path (from the observation of real system or single-model simulation) and to use it to construct perturbed sample-paths. The perturbed path is the result of injecting variations of decision variables into the system. Suppose that during the experiment time *T*, a total of $L_{i}$ parts are served by machine *i*. Then the production rate of machine *i* in the nominal path is:

(9) $$PR_{i}=L_{i}/T$$

Production lines are considered as Discrete-EventDynamic Systems in which perturbations do not only affect the events times and durations, but also the number of parts served by the different machines, resulting in the increase or the decrease of blocking and/or starvation periods. Let $\Delta t_{i}$ be the total event time perturbation on machine *i* at the end of a simulation replication. The production rate of machine *i* in the perturbed path is:

(10) $$PR_{i}^{\prime }=L_{i}/(T+\Delta t_{i})$$

**Figure 3.**
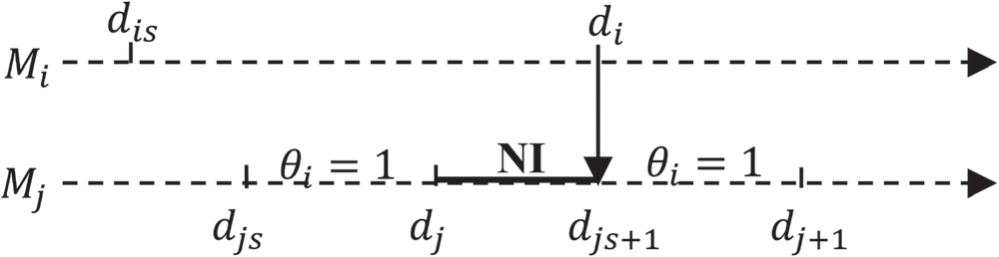
Sample path with NI case.

$PR_{i}$ is derived from the nominal path observation, and $PR_{i}^{\prime }$ is calculated through the application of perturbation generation and propagation rules on the nominal path. Since perturbation generation rules are parameter specific, it is related here to the variation of buffer capacities and service times. As buffer capacities are discrete variables, a simple increase of the size could lead to a highly perturbed path with important changes in the order of the events, which does not reflect the system under consideration. Therefore, Infinitesimal Perturbation Analysis (IPA) cannot be considered (Glasserman 1991; Suri 1987). We consider thus the development of FPA as an extension of PA when perturbations are finite (large amplitude) and where the system's performance is not affected by the order changes in events (Ho 1987; Suri and Leung 1989). Indeed, we assume that the type of future interactions that a perturbation, once introduced, can encounter and/or lead through the system in the perturbed path in and the nominal path is statistically similar (Ho 1987). Therefore, the nominal path can be used to determine the response of the system to a perturbation. This shows the advantage provided by this technique compared to the usual techniques of gradients estimates. Let $d_{i}$ be the end time of the service duration on machine *i,* and $d_{is}$ be the nominal service start times of the event under consideration. Figure 3 shows a case where the machine $M_{j}$ is NI (starved) in the nominal path, ended by a part transfer from $M_{i}$ to $M_{j}$ (the arrow indicates the direction of passage of the release part from one machine to another). To determine how the system may evolve in the perturbed environment, we assume that both $M_{i}$ and $M_{j}$ encounter the perturbation $\Delta t_{i}$ and $\Delta t_{j}$ (generated or propagated), respectively. The total perturbation value propagated on $M_{j}$ is calculated through the formula:

(11) $$\begin{array}{rl} \Delta t_{j}^{\prime } & =(\text{new starvation end time})\\ & \;\text{--}\;(\text{old starvation end time}) \end{array}$$

**Figure 4.**
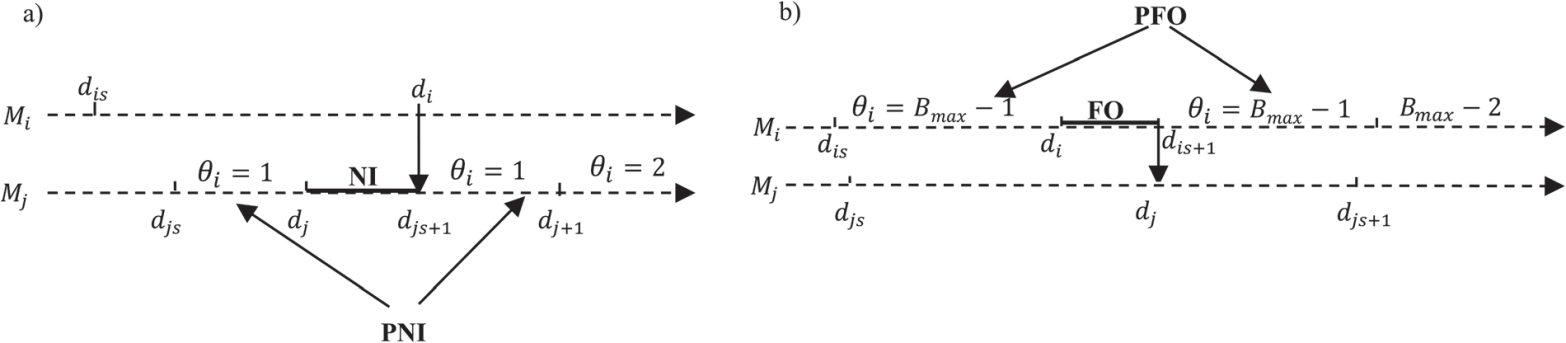
PNI and PFO periods.

We also define a potential starvation (resp. blocking) PNI (resp. PFO) on a server $M_{i}$ as a state in which its upstream (resp. downstream) buffer contains only one (resp. $B_{\text{max}}-1$) unit(s). When a server is starved (resp. blocked), it is no longer able to receive or to deliver, and no routing of parts can be possible (see Figure 4a,b, respectively).

#### 4.2.1. Case of starvation (NI)

Consider the nominal event path in Figure 4a. After interaction between the two sequences of perturbed events of $M_{i}$ and $M_{j}$, two cases can occur.

*Case 1:* $d_{i}+\Delta t_{i}>d_{j}+\Delta t_{j}$. The NI period still exists in the perturbed path. The perturbation on $M_{i}$ is fully propagated to $M_{j}$. The new value of the perturbation resulting on $M_{j}$ is:

(12) $$\Delta t_{j}^{\prime }=(d_{i}+\Delta t_{i})-d_{i}=\Delta t_{i}$$

*Case 2:* $d_{i}+\Delta t_{i}\leq d_{j}+\Delta t_{j}$. The NI period is eliminated and replaced by a PNI period. The perturbation is partially propagated, and the new perturbation generated is then:

(13) $$\Delta t_{j}^{\prime }=(d_{j}+\Delta t_{j})-d_{i}=\Delta t_{j}+(d_{j}-\;d_{i})$$

Note that the term ( $d_{i}-d_{j}$) is negative, attenuating the effect of the perturbation $\Delta t_{j}$ on $M_{j}$, but it does not make it possible to obtain a value of the new negative perturbation $\Delta t_{j}^{\prime }$.

#### 4.2.2. Case of potential starvation (PNI)

**Figure 5.**
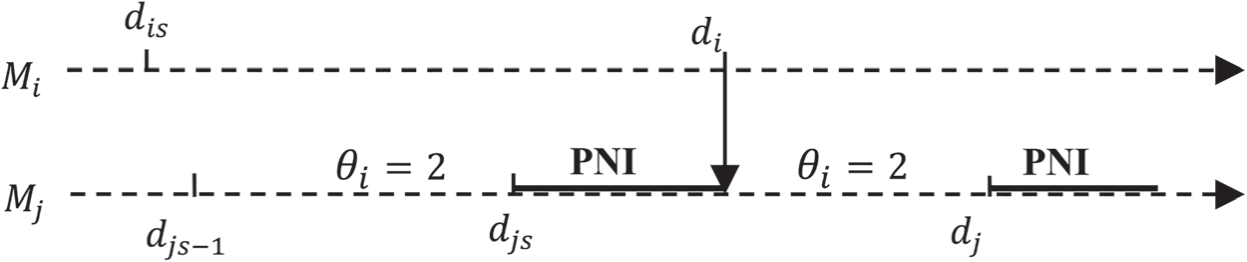
Sample path with PNI case.

Consider Figure 5, where the machine $M_{j}$ is in a PNI state in the nominal path due to the unique part existing in the buffer $\theta_{i}$.

Interaction between servers may be transformed into real NI in the perturbed path and two cases can be distinguished.

*Case 1:* $d_{i}+\Delta t_{i}\leq d_{j}+\Delta t_{j}$. The PNI remains PNI, and all service event sequences retain their perturbations:

(14) $$\begin{array}{rl} \Delta t_{i}^{\prime } & =\Delta t_{i} \end{array}$$

(15) $$\begin{array}{rl} \Delta t_{j}^{\prime } & =\Delta t_{j} \end{array}$$

*Case 2:* $d_{i}+\Delta t_{i}>d_{j}+\Delta t_{j}$. In the perturbed path, the part transferred from $M_{i}$ arrives later than the end of the service of the last part contained in the stock and thus in use by $M_{i}$. This situation creates a starvation period during which $M_{j}$ remains idle. The PNI is transformed into a starvation period on $M_{j}$. The perturbation is then partially propagated, creating a NI period on $M_{j}$. Note that ( $d_{i}-d_{j}$) is negative, which asserts the phenomenon of partial propagation.

(16) $$\Delta t_{j}^{\prime }=(d_{i}+\Delta t_{i})-d_{j}=\Delta t_{i}+(d_{i}-d_{j})$$

All cases of NI or PNI periods can be summarised in the following equation, where [NI] denotes the algebraic value of the duration of a starvation interval for the machine $M_{j}$, i.e. $\text{NI}=(d_{i}-d_{j})$.

(17) $$\Delta t_{j}^{\prime }=\text{max }\{\Delta t_{i}\text{ + }[\text{NI}],\;\Delta t_{j}\}-\text{max}\{\text{0,}[\text{NI}]\}$$

#### 4.2.3. Case of blocking (FO)

The development of the perturbation propagation rules in the case of blocking (Figure 4b) is treated similarly to the case of starvation.

#### 4.2.4. Case of potential blocking (PFO).

**Figure 6.**
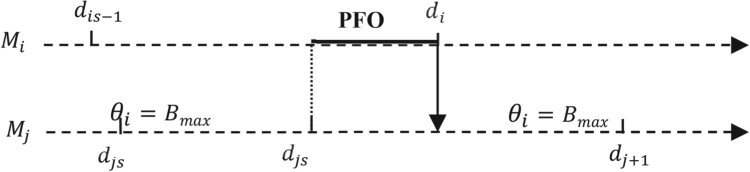
Sample path with PFO case.

Consider Figure 6 where the machine $M_{j}$ is in a PFO period in the nominal path.

Two cases can be presented in the perturbed path.

*Case 1:* $d_{i}+\Delta t_{i}\geq d_{j}+\Delta t_{j}$. The PFO still exists in the perturbed path of $M_{i}$. There is no upstream propagation, and each machine keeps its already acquired perturbations.

(18) $$\begin{array}{rl} \Delta t_{i}^{\prime } & =\Delta t_{i} \end{array}$$

(19) $$\begin{array}{rl} \Delta t_{j}^{\prime } & =\Delta t_{j} \end{array}$$

*Case 2:* $d_{i}+\Delta t_{i}<d_{j}+\Delta t_{j}$. The potential FO is transformed into a blocking period on $M_{i}$. The perturbation is partially propagated from $M_{j}$, creating thus a FO period on $M_{i}$:

(20) $$\Delta t_{i}^{\prime }=(d_{j}+\Delta t_{j})-d_{i}=\Delta t_{j}+(d_{j}-d_{i})$$

All cases of FO or PFO periods can be summarised in the next equation, where [FO] is the algebraic value of the duration of a blocking interval for the machine $M_{i}$, i.e. $\text{FO}=(d_{i}-d_{j})$.

(21) $$\Delta t_{i}^{\prime }=\text{max }\{\Delta t_{j}\text{ + }[\text{FO}],\;\Delta t_{i}\}-\text{max}\{\text{0,}[\text{FO}]\}$$

### 4.3. FPA algorithm

#### 4.3.1. FPA algorithm for buffer capacities

Consider an observation (or a simulation) of a production line, on which a total of L parts are treated within a time duration *T*. We assume that all possible events during the simulation run can occur (i.e. the simulation is sufficiently long). The PR calculated over the duration *T* is approximated by the Equation (22):

(22) PR=L/T

Hence,

(23) $$\partial PR/\partial \theta_{i}=\partial \left(\frac{L}{T}\right)/\partial \theta_{i}$$

Assuming that the stopping criterion of the simulation is the total number of parts served by the output machine of the system (i.e. *L*), we have

(24) $$\begin{array}{rl} \frac{\partial PR}{\partial \theta_{i}} & =\frac{\partial PR}{\partial T}\cdot \frac{\partial T}{\partial \theta_{i}}=-\left(\frac{L}{T^{2}}\right)\cdot \frac{\partial T}{\partial \theta_{i}}\\ & =-\left(\frac{L}{T}\right)\cdot \left(\frac{1}{T}\right)\cdot \frac{\partial \text{T}}{\partial \theta_{i}}=-\frac{PR}{T}\cdot \frac{\partial \text{T}}{\partial \theta_{\text{i}}} \end{array}$$

where $\partial T/\;\partial \theta_{i}$ denotes the total perturbation time that affects the system when a perturbation $\Delta \theta_{i}$ is introduced. In this study, the perturbation is one unit stock capacity related to each relevant event. The prediction of the impact of the perturbation concerns the event's persistence in the perturbed path and its duration. Thanks to the propagation rules of FPA, this incrementation of one unit is virtual and used only to evaluate the PR gradient and the value of $\partial T/\;\partial \theta_{i}$ as follows:

(25) $$\frac{\partial PR}{\partial \theta_{i}}=-\frac{PR}{T}\cdot \frac{\partial T}{\partial \theta_{i}}=-\frac{PR}{T}\cdot \Delta t_{n}$$

where $\Delta t_{n}$ represents the total temporal perturbation acquired by the output machine $M_{n}\;$after the treatment of L parts, which is also the total time gain (or loss) that is cumulated at the output machine $M_{n}$. In other ways, $\Delta t_{n}$ is an estimator of $\frac{\partial T}{\partial \theta_{i}}$. Algorithm 2 is a simplified FPA algorithm for buffer capacities.

**Table:** 

**Algorithm 2:** FPA for buffer capacities
**Generation of perturbations**
**Step 1**
** For** i=1 to n−1, **do:** $\text{Su}{\text{m}}_{i+1}=0$; Select $\theta_{i}$ (initialisation of accumulators $Sum_{i+1}$)
**Step 2**
** For ‘** $M_{i}(t)$ is FO for the first time ‘, **do:** $\theta_{i}=\theta_{i}+\Delta \theta_{i};$ $\text{Su}{\text{m}}_{i}=t_{i}\cdot \Delta \theta_{i}$
** **( $\theta_{i}$ is the buffer size located between two consecutive machines $M_{i}$ and $M_{i+1}$; in this paper $\Delta \theta_{i}=1$)
Propagation of perturbation
**Step 3.a**
** For** any event ‘ $M_{j}(t)$ is NI ‘ (resp. PNI), **do:** $\text{Su}{\text{m}}_{j}=\max\{\text{Su}{\text{m}}_{i}+[\text{NI}],\text{Su}{\text{m}}_{j}\}-\max\{0,[\text{NI}]\}$
**Step 3.b** (does not apply at the first variation of the stock)
**For** any event ‘ $M_{i}(t)$ is FO ‘ (resp. PFO), **do:** $\text{Su}{\text{m}}_{i}=\max\{\text{Su}{\text{m}}_{j}+[\text{FO}],\text{Su}{\text{m}}_{i}\}-\max\{0,[\text{FO}]\}$
**Step 4**
** If** I = n (last machine), **then**
** ** $\frac{\partial \text{PR}}{\partial \theta_{i}}=-\left(\frac{\text{PR}}{T}\right)\cdot \text{Su}{\text{m}}_{n}$
** ** $\text{P}{\text{R}}^{*}=\frac{P}{T-\text{Su}{\text{m}}_{n}}$ ( $PR^{*}$denotes the estimated production rate in the perturbed trajectory)
** STOP**
** Else** Go to Step 1

#### 4.3.2. FPA algorithm for service times

To estimate $\partial \text{PR}/\partial \theta_{i}$ (for i=1,…,n), we use the perturbation analysis gradient estimator. Algorithm 3 is a slightly modified version of the method presented by Suri and Leung (1987). The first step is the initialisation of accumulators $A_{ij}$ ( i,j=1,…,n), where $A_{ij}\;$are separate accumulators needed for the gradient calculations. So, at each event (end of operation on a machine), the value of the gradient is calculated and $\frac{dt_{i}}{d\theta_{i}}$ is added to $A_{ij}$. $\frac{dt_{i}}{d\theta_{i}}$ denotes the sample gradient of the variable $t_{i}$, where $\frac{dt_{i}}{d\theta_{i}}=1$ since in this work $t_{i}=\theta_{i}$ (Step 2). At the end of the simulation, an estimation of $\partial \text{PR}/\partial \theta_{i}$ is given by the following equation, where $A_{ni}$ denotes the value of accumulator at the end of the simulation.

(26) $$\partial \text{PR}/\partial \theta_{i}=-(\text{PR}/T)\cdot A_{ni}$$

**Table:** 

**Algorithm 3:** FPA for service times
**Step 1** *Initialisation of accumulators* $A_{ij}$
** ** $A_{ij}=0$ for i,j=1,…,n *(* $A_{ij}$ *are the accumulators used to calculate the gradients)*
**Step 2** At the end of an operation on machine *i* with a service time $t_{i}$
** ** $A_{ii}=A_{ii}+\frac{dt_{i}}{d\theta_{i}}$
** If** a part that is leaving machine *i* going to machine *m* ends at period *NI* on machine *m,* **then**
** ** $A_{mj}=A_{ij}$ j=1,…,n ** If** a part leaving machine *i* going to machine *m* ends a period and is *FO* on machine *i,* **then**
** ** $A_{ij}=A_{mj}$ j=1,…,n
**Step 3**
$\partial PR/\partial \theta_{i}=-(PR/T)\cdot A_{ni}$

### 4.4. Stochastic algorithm (SA)

The gradient estimates resulting from FPA are injected into a SA to compute the (near-)optimal allocation of buffer capacities and service times for the production line. The SA presented in Algorithm 4 is based on Robbins and Monro (1951), where the gradients are considered from the PR function's projection on the constraint hyperplane $\underset{i}{\sum }\theta_{i}$ =  constant. The SA updates intermediate variables $\theta_{i}$ (buffer capacities and service times) at each iteration corresponding to a perturbed path generation, following the direction of the gradient. Here, a single run optimisation algorithm is used, in the sense that the update procedure is activated at each end service of *p* units (p<L). The value of *p* is fixed empirically. The update procedure of the configuration uses the gradients calculation's technique based on FPA (Step 3). Also, we chose $a_{k}$ (respectively $b_{k}$) as numerical suites of type α/k (respectively β/k), where α (resp. β) is a random constant with a chosen initial value such that the value of the $\theta_{i}$ after each iteration are kept in the same order. Their values determine the step size of the production rate. A precaution is proposed in Step 4 if some updated values of $\theta_{i}^{k+1}$ are negative. Step 5 returns the final variable values where a discretisation of buffer capacities values is necessary. The algorithm stops when a (near-)optimal solution is obtained. A trade-off between the accuracy of the estimated parameter and the simulation length is acquired by the parameter ϵ, set here to $10^{-4}$. The benefit of the stopping criterion used is that it considers simultaneously the variations of all variables $\theta_{i}$. In other words, the algorithm stops when the variation of the variables becomes negligible within the same iteration.

**Table:** 

**Algorithm 4:** Stochastic Algorithm (SA)	
**Initialisation** k = 1	
**Step 1 **Choose initial values of $\theta_{i}^{k}($for i=1,…,2n−1)	
**Step 2 **Simulate *p* (< L) parts for $\theta_{i}^{k}$ and estimate $\frac{\partial PR}{\partial \theta_{i}}$ using FPA	
**Step 3 ** $\theta_{i}^{k+1}=\theta_{i}^{k}+a_{k}\left(\partial PR/\partial \theta_{i}-\frac{1}{n-1}\overset{n-1}{\underset{i=1}{\sum }}\partial PR/\partial \theta_{i}\right)$	(buffer capacities)
** ** $\theta_{i}^{k+1}=\theta_{i}^{k}+b_{k}\left(\partial PR/\partial \theta_{i}-\frac{1}{n}\overset{2n-1}{\underset{i=n}{\sum }}\partial PR/\partial \theta_{i}\right)$	(service times)
**Step 4 If ** $\theta_{i}^{k+1}$ ≤ 0, **then**	
** ** $\theta_{p}^{k}$ = Arg min $\theta_{i}^{k}$; $\theta_{p}^{k+1}$ = Arg min $\theta_{i}^{k+1}$	
** ** $a∼U(\text{0,1});h=\left|\frac{a\cdot \theta_{p}^{k}}{\theta_{p}^{k+1}-\theta_{p}^{k}}\right|$	
** ** $\theta_{i}^{k+1}=\theta_{i}^{k}+h\cdot a_{k}\cdot \left(\frac{\partial PR}{\partial \theta_{i}}-\frac{1}{n-1}\overset{n}{\underset{i=1}{\sum }}\frac{\partial PR}{\partial \theta_{i}}\right)$	(buffer capacities)
** ** $\theta_{i}^{k+1}=\theta_{i}^{k}+h\cdot b_{k}\cdot \left(\frac{\partial PR}{\partial \theta_{i}}-\frac{1}{n}\overset{n}{\underset{i=1}{\sum }}\frac{\partial PR}{\partial \theta_{i}}\right)$	(service times)
**Step 5 If ** $|\theta_{p}^{k+1}-\theta_{p}^{k}|\leq \epsilon ,$ **then**	
** ** $\theta_{i}$ = Anint $\left(\theta_{i}^{k}\right)$; i=1,…,n−1	(discretisation of buffer capacities)
	(Anint: an Arena operator that takes the near integer value)
** ** $\theta_{i}=\theta_{i}^{k}$; i=n,…,2n−1	(service times)
** STOP**	
** If** *p* parts are treated, **then STOP**; **Else** k = k + 1 and Go to Step 2	

## 5. Experiments

Since one of the goals of the paper is to identify design principles, guidelines, and patterns that could help production managers in determining the optimal values for buffer sizes and service times, the experiments are designed to reach this goal. We consider unreliable production lines with a number n of machines ranging from 3 to 100 (for the case of reliable machines, see for instance Spinellis, Papadopoulos, and Smith 2000). Not only the PR values reached by the method proposed are compared with the ones obtained by other techniques from the literature, but also the obtained solution patterns are compared with the ones from other studies. Several experiments are conducted: (i) small instances have n = 3, 5 and 7 machines; (ii) medium/large instances have n = 10, 20 and 40 machines; and (iii) very large instances have n = 50, 75 and 100 machines. The algorithms are implemented in Java and the models are developed by using the simulation language Arena V14.0 (Altiok and Melamed 2010). The experiments are run on intel Core (TM) i5 CPU @ 1.9-GHz with 8 GB of RAM. In all cases, the PR average is calculated by simulating 10,000 parts with 20 runs/replications. The total service time $T_{max}$ is 3*N* time-units for each machine. All machines are identical and are subject to breakdown. Except if other information is provided, for all the instances, the repair and failure times are geometrically distributed with MTTR = 10 and MTBF = 70, respectively. GA uses tournament selection, arithmetic crossover operators, and a population size of 30. The algorithm stops when it reaches the maximum fixed number of 20 generations or when achieving (at a given generation) a PR better than the best PR ever reached. The computation times (presented in the last column of Tables 3, 4 and 7) are not discussed in detail, but the order of magnitude is 84 (resp. 160) minutes on average for large (resp. very large) production lines, which is reasonable from a practical standpoint with respect to various studies in production (e.g. Respen, Zufferey, and Amaldi (2016), Zufferey (2016)).

In Subsection 5.1, to measure the benefit of combining GA with FPA, we compare the two latter methods (used independently) with GA-FPA. In Subsection 5.2, we measure the benefit of optimising simultaneously buffers and service times. In Subsection 5.3, experiments are conducted to identify the service-time and buffer allocation patterns of the GA-FPA solutions. In Subsection 5.4, a sensitivity analysis is proposed with respect to having a machine with a different repair time or with a different failure time. Finally, in Subsection 5.5, the performance of the proposed hybrid approach is investigated for the very large production lines.

### 5.1, Comparison of GA, PA, and GA-FPA

GA-FPA is compared with FPA and GA (considered independently). Table 1 gives the results for instances with n in 5, 10, 15, 20, 40. Columns 1 and 2 present n and $B_{\text{max}}$, respectively. Next, from columns 3–5, the average PR is given for GA, FPA, and GA-FPA, respectively. In terms of solution quality, GA-FPA achieves the best solutions for each instance.

**Table 1.** Results of GA, FPA and GA-FPA.

Parameters		Average PR		
*N*	*B*_max_	GA	FPA	GA-FPA
5	20	0.248063	0.246847	0.248957
10	45	0.233728	0.224865	0.235617
15	70	0.227555	0.218695	0.228398
20	95	0.223371	0.211147	0.226174
40	390	0.230927	0.220034	0.238537

**Figure 7.**
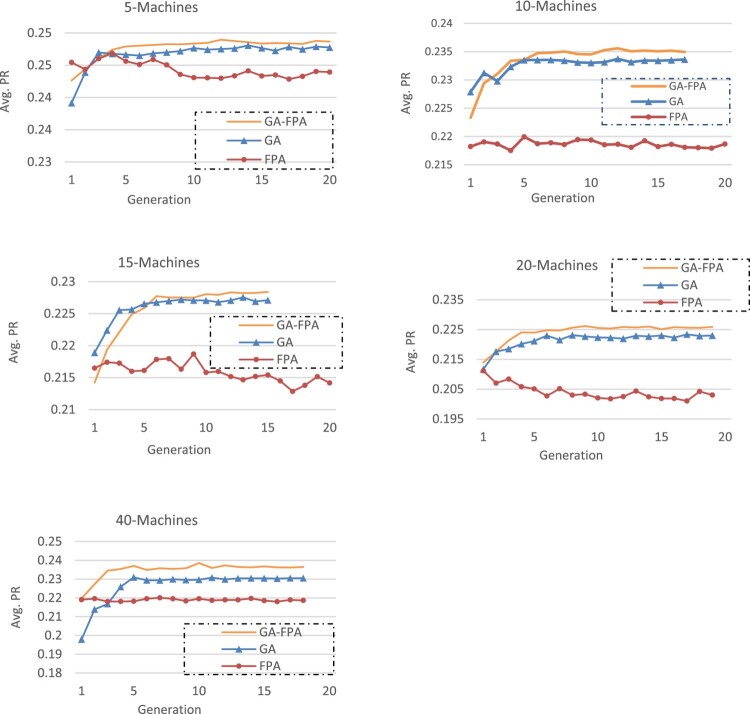
Convergence behaviour of the proposed methods.

The evolutions of the average PR with respect to the number of generations for the five instances are presented in Figure 7. Regarding the convergence behaviour, only a few generations are needed for GA-FPA to converge to its best solutions (it is not the case for GA and FPA). We can explain this efficient convergence of GA-FPA by the exploration ability of its GA feature to quickly identify promising regions of the solution space, and by the exploitation ability of its FPA component to intensify the search in such promising regions.

### 5.2. Benefit of optimising simultaneously buffers and service times

To measure the benefit of the simultaneous allocation of buffers and service times, an idea is to compare the results of the simultaneous allocation with those of the buffer allocation and the service time allocation considered alone. In the case of buffer-capacity optimisation, the service times are all equal and satisfy the $T_{\text{max}}$ constraint. Whereas in the case of service-time optimisation, the buffer capacities are as equal as possible, with some balanced adjustments to have integer numbers and to satisfy the $B_{\text{max}}$ constraint.

Table 2 presents the results for instances with n in 5, 10, 15, 20, 40 machines. The first two columns present the instance parameters, followed by the average PR for service time allocation, buffer allocation, and simultaneous allocation. Note that for the instance with n = 40, only five runs (instead of 20) have been carried out.

**Table 2.** Results of service time, buffer, and simultaneous allocations.

Parameters		Average PR		
		Service time	Buffer	Simultaneous
*N*	*B*_max_	allocation	allocation	allocation
5	20	0.245621	0.247458	0.248957
10	45	0.234046	0.235018	0.235617
15	70	0.226720	0.227835	0.228398
20	95	0.225672	0.225837	0.226174
40	390	0.237693	0.237696	0.238537

We can easily observe that the joint optimisation of buffers and service rates obtains the best average PR results for all instances. The quantification of this benefit may help practitioners and scientists for designing and planning production lines.

### 5.3. Solution patterns obtained by GA-FPA

Tables 3 and 4 present experiments with n in 3, 5, 7 for the small instances (Table 3), and with n in 10, 12, 15, 20, 40 for the medium/large instances (Table 4). $B_{\text{max}}$ is given in the second column. To investigate the buffer allocation scheme with respect to a uniform allocation, $B_{\text{max}}$ is chosen to be a multiple of the number of buffers. Column 3 (resp. 4) presents the best buffer capacities $b^{*}$ (resp. service times $t^{*}$) returned by GA-FPA. Note that for Table 4, due to the large number of indicated values, both $b^{*}$ and $t^{*}$ are presented in column 3 (for n > 12). Computing times are given in the last column, in seconds.

**Table 3.** Results for small production lines (involving 3, 5, and 7 machines).

*N*	$B_{\text{max}}$	$b^{*}$	$t^{*}$	CPU(s)
3	10	5 5	3.02 2.96 3.02	977
	20	10 10	3.01 2.97 3.02	1132
	30	15 15	3.02 2.98 3.00	1191
	40	20 20	3.01 2.98 3.01	805
5	20	5 6 5 4	3.02 3.02 2.99 2.98 2.99	2506
	40	10 11 10 9	3.04 2.99 2.98 2.99 3.01	1463
	60	14 17 16 13	3.05 3.00 2.97 2.98 3.00	2018
	80	19 22 20 19	3.05 2.99 3.00 2.99 3.00	1469
7	30	3 5 7 7 6 2	3.03 3.02 2.96 3.00 2.98 3.00 3.03	2828
	60	9 10 11 11 10 9	3.02 2.99 2.98 3.00 3.00 2.99 3.02	2599
	90	15 15 16 16 14 14	3.04 2.98 3.00 2.98 3.00 2.97 3.03	1874
	120	20 20 20 21 20 19	3.02 2.97 3.00 2.99 3.01 2.99 3.02	2696

**Table 4.** Results for medium/large production lines (involving 12, 15, 20, and 40 machines).

*N*	$B_{\text{max}}$	$b^{*}$	$t^{*}$	CPU (s)
**10**	45	4 5 5 5 5 6 5 5 5	3.02 3.01 2.97 3.01 3.00 2.98 3.00 2.98 3.02 3.01	2183
	90	10 11 9 9 10 11 11 9 10	3.02 2.97 3.03 2.99 3.00 3.00 2.99 2.97 3.03 3.00	2249
	135	15 17 15 15 14 16 16 14 15	3.03 2.99 2.98 3.01 3.00 2.99 3.00 2.99 3.00 3.02	2353
	180	19 21 18 22 21 21 20 19 19	3.04 2.99 2.97 3.00 2.98 3.01 2.99 2.99 2.98 3.04	2269
***N***	$B_{\text{max}}$	$b^{*}$	$t^{*}$	
**12**	55	4 4 6 6 4 5 5 6 6 5 4	3.01 2.99 2.98 2.96 3.00 3.01 3.02 2.97 3.00 3.01 3.01 3.04	2219
	110	10 10 9 10 10 10 11 10 10 11 9	3.02 2.99 3.00 3.01 2.97 2.99 3.01 2.99 3.01 2.97 3.01 3.02	2219
	165	15 15 16 16 16 16 14 13 14 15 15	3.00 2.99 3.03 2.99 2.98 3.02 2.99 2.98 3.00 3.00 3.01 3.01	2591
	220	20 20 21 19 21 19 21 21 19 20 19	3.00 3.02 2.99 3.01 3.02 2.99 2.96 3.00 3.00 2.99 3.00 3.02	3378
***N***	$B_{\text{max}}$	$b^{*}$		
**15**	70	4 4 5 5 6 5 5 5 6 6 5 5 5 4		2806
	140	10 10 8 10 12 10 10 11 11 10 9 10 10 9		3118
	210	14 15 14 15 16 16 16 15 16 15 14 15 15 14		2647
	280	20 20 18 22 20 22 21 20 19 20 19 20 20 19		2426
	$B_{\text{max}}$	$t^{*}$		
	70	3.00 3.03 2.97 3.03 2.98 2.99 3.01 2.96 3.01 3.02 3.01 2.99 3.00 3.00 3.00		
	140	3.00 3.01 3.03 3.01 3.01 3.00 3.01 2.98 3.02 3.02 2.98 2.98 2.97 2.99 3.00		
	210	2.99 3.03 3.01 3.01 3.01 2.98 2.98 3.01 2.98 3.02 2.99 2.98 2.98 3.02 3.01		
	280	3.03 2.99 3.00 2.99 3.01 3.00 2.98 3.02 3.01 2.97 3.01 2.98 3.00 3.00 3.01		
***N***	$B_{\text{max}}$	$b^{*}$		
**20**	95	4 5 5 6 5 4 5 7 5 4 5 6 6 4 4 5 7 5 3		4921
	190	10 9 10 9 10 10 10 9 9 12 10 11 10 10 9 11 12 9 10		5061
	285	15 16 14 16 13 15 16 16 17 15 15 15 14 16 14 14 14 15 15		2928
	380	20 22 21 20 21 21 20 20 19 20 20 20 21 19 19 19 19 20 19		3558
	$B_{\text{max}}$	$t^{*}$		
	95	3.02 2.97 2.97 3.01 3.02 3.00 3.04 3.00 3.02 2.98 3.00 2.99 2.96 3.03 3.02 3.00 2.95 2.99 3.01 3.02		
	190	3.02 3.01 3.02 3.00 2.98 2.98 3.00 2.98 3.00 3.00 3.00 3.01 2.96 3.02 2.97 2.99 3.05 3.02 2.98 3.01		
	285	3.01 3.01 2.99 3.02 3.03 3.02 3.04 2.97 3.00 2.98 2.97 3.00 2.99 2.97 3.01 3.02 3.01 2.99 2.98 2.99		
	380	3.02 2.99 3.01 2.98 3.03 3.02 2.97 2.99 3.04 3.04 2.98 2.99 2.99 2.99 2.99 3.03 2.95 3.02 2.98 2.99		
***N***	$B_{\text{max}}$	$b^{*}$		
**40**	195	5 5 6 6 5 6 4 4 6 6 4 6 4 6 5 5 5 5 5 6		5759
		5 5 6 4 5 5 6 4 4 5 5 4 4 6 5 5 4 5 4		
	390	10 10 9 10 9 10 11 10 10 10 10 11 11 9 11 9 10 10 10 9		5101
		9 10 10 9 9 11 12 10 10 10 11 9 9 9 11 10 11 12 9		
	585	15 16 16 14 15 14 16 14 15 16 15 15 15 14 14 17 15 15 16 15		4179
		15 15 14 17 14 15 14 14 15 17 15 14 15 14 15 16 14 15 15		
	780	19 21 18 20 20 19 19 22 20 19 22 21 20 23 19 19 19 21 20 21		5166
		20 20 20 20 21 19 20 18 20 20 21 20 20 20 20 20 20 20 19		
	$B_{\text{max}}$	$t^{*}$		
	195	2.98 3.02 2.98 3.02 3.01 3.02 2.98 3.03 3.02 2.98 3.01 3.00 2.98 2.98 3.00 3.01 3.00 3.00 3.02 2.99		
		3.01 3.02 2.98 3.03 3.02 2.99 2.97 2.99 3.02 3.00 3.00 3.00 2.98 2.97 3.00 3.01 2.99 3.01 2.99 2.99		
	390	3.03 2.98 2.97 2.98 2.97 2.98 3.02 3.02 2.99 3.00 3.02 2.96 3.01 3.01 2.98 2.98 3.01 2.99 3.01 2.98		
		2.99 3.01 3.05 3.00 3.02 3.03 3.06 2.98 3.03 3.00 3.00 2.99 2.97 2.99 2.99 2.98 3.01 2.98 3.01 3.02		
	585	2.98 2.97 3.00 2.99 3.00 2.97 3.00 3.02 2.97 3.01 3.00 3.00 2.98 3.00 2.97 2.98 2.99 3.01 3.02 2.98		
		3.00 3.03 3.03 3.03 3.02 2.96 2.97 2.96 3.00 3.00 3.03 2.98 3.01 3.01 3.01 3.02 3.05 3.00 3.03 3.02		
	780	3.02 2.97 2.98 3.00 3.03 2.96 3.01 3.00 3.00 3.00 2.96 2.99 3.00 2.99 3.00 3.03 3.00 3.01 2.99 2.96		
		3.03 3.01 3.01 3.03 3.00 2.96 2.99 3.01 3.02 3.03 3.00 2.98 2.98 2.98 3.03 3.01 2.99 3.03 2.99 3.02		

**Figure 8.**
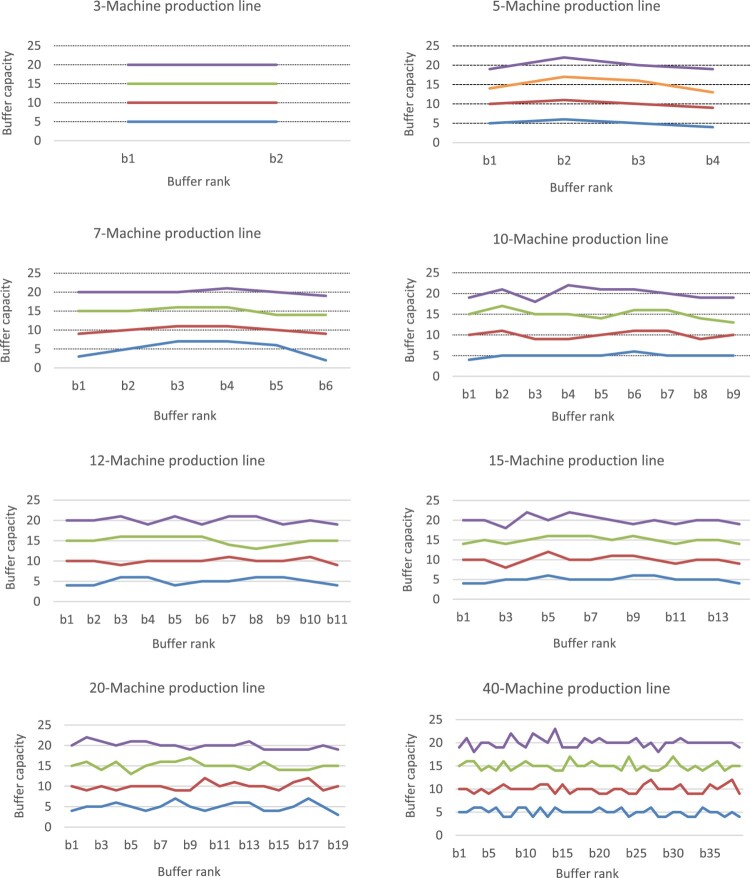
Allocation patterns for buffer capacities.

**Figure 9.**
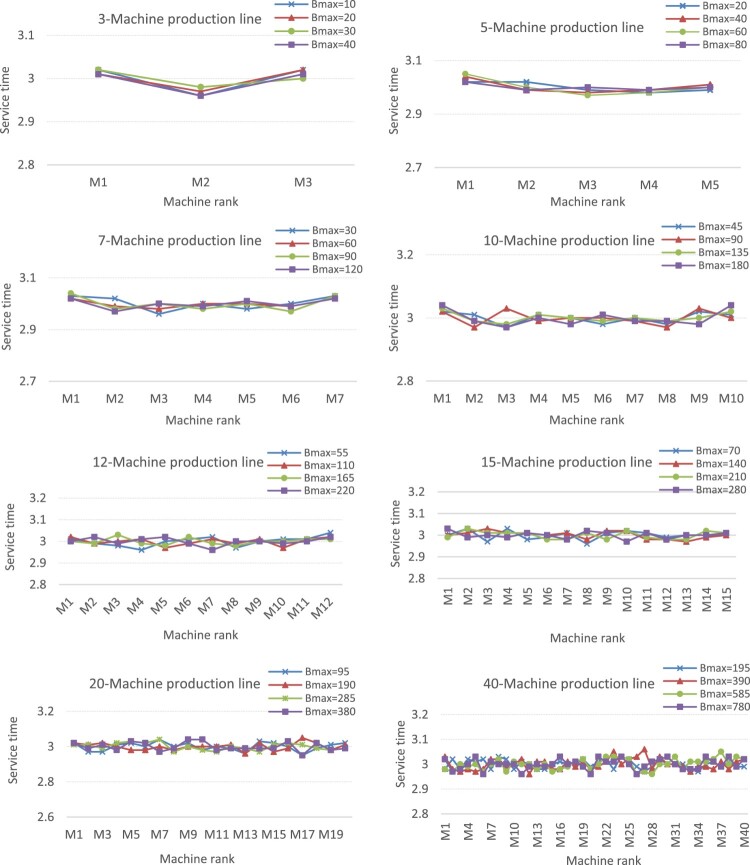
Allocation patterns for service times.

Figure 8 provides the allocation patterns for buffer capacities throughout the available buffer spaces between machines. Eight cases are presented (three cases of small-size production lines with n in 3, 5, 7, five cases of medium/large-size production lines with n in 10, 12, 15, 20, 40). For each case, four values of $B_{\text{max}}$ are tested. Likewise, Figure 9 presents the allocation patterns for the service times of the machines. Note that since we determine the capacity of different buffers (Figure 8) and the service time of different machines (Figure 9), we could have represented these allocations as clouds of points. However, for sake of clarity, we prefer to connect the different points and consider a representation as if it was a continuous function.

For the small instances, the results for the allocation of buffer capacities (b* values of Tables 3 and 4, and patterns of Figure 8) show that there is more storage space needed in the middle of the production line and less storage space needed at the end. These pattern allocations are likely to facilitate parts flowing downstream and to avoid possible blockages. For the allocation of service times, the results (t* values of Tables 3 and 4, and patterns of Figure 9) show that the machines at the end of the line need more processing time than the machines at the centre. This is certainly to facilitate the passage of parts throughout the production line and thus to avoid congestion at the centre of the line. These results corroborate the findings of Smith (2018) and Hillier and Hillier (2006). In their studies, the authors mentioned this service-time (resp. buffer-capacity) allocation pattern as bowl-shaped (resp. inverted bowl-shaped) allocation. The bowl-shape name arises from the fact that the service-time values of the first and the last machine are larger than the service times of other machines which are relatively close together.

For the medium/large instances (i.e. from n = 10), the results show that some buffers in the middle of the line require lesser storage capacities than the buffers at the ends of the production line. This buffer allocation pattern (inverted bowl-shaped) is less accentuated as the number *N* of machines increases. Indeed, the uniform allocation of buffers becomes more pronounced, as shown in Figure 7. It can also be seen that most cases favour the allocation of slightly low storage capacities at the end of the production line. This pattern allocation of buffers is likely to facilitate parts flowing downstream and thus to avoid any blockages. Regarding the allocation of service times, the results with n = 10 show that the service times of the first and the last machine are slightly larger than the service times of other machines (which are still relatively close together), and some machines in the middle of the line need service times relatively like those of the end. Figure 8 shows that the bowl-shaped times-allocation patterns tend to disappear as *N* increases. Instead, a uniform allocation tends to persist. These results are similar to the findings of Spinellis, Papadopoulos, and Smith (2000) considering the buffer allocation. However, as far as the service time is concerned, the service time allocation does not follow a uniform allocation but diminishes towards the end of the line.

In summary, the inverted bowl-shaped pattern for buffer capacities and the bowl-shaped allocation for service times seem to be diminished as the number of machines of the production line increases, and instead, both the service times and buffers allocations tend to follow roughly a uniform allocation.

### 5.4. Sensitivity analysis

Two series of experiments are presented to determine whether the allocation patterns found when all machines have the same failure and repair times are respected or, otherwise, whether there are specific buffer and service time allocation patterns when the failure and repair rates are modified. Here, the PR of the production line is calculated by treating 10,000 parts for 10 runs/replications. The results are reported in Table 5 and 6 for instances with n in 3, 10, 20, where the total buffer size $B_{max}$ to be assigned is presented in the second column. The fourth and fifth columns present the returned buffer capacities $b^{*}$ and service times $t^{*}$, respectively.

**Table 5.** Results if one machine has a different repair time.

N	B	Case	$b^{*}$									$t^{*}$									
3	40	1	28					12				2.63			3.16			3.21			
		2	19					21				3.13			2.68			3.19			
		3	15					25				3.21			3.17			2.62			
10	180	1	21	21	21	19	18	21	19	20	20	2.48	2.94	3.05	3.08	3.08	3.08	3.10	3.08	3.05	3.06
		2	21	22	21	21	18	19	19	19	20	2.99	2.50	2.97	3.11	3.10	3.10	3.07	3.08	3.03	3.05
		3	21	20	22	19	20	20	19	20	19	3.13	2.99	2.45	2.96	3.04	3.08	3.10	3.09	3.09	3.07
		4	20	18	21	22	20	21	19	19	20	3.14	3.09	2.96	2.42	2.95	3.06	3.10	3.10	3.09	3.09
		5	20	21	19	22	20	21	20	20	17	3.18	3.15	3.08	2.95	2.42	2.98	3.02	3.06	3.09	3.07
		6	20	21	21	20	20	21	19	18	20	3.15	3.20	3.10	3.09	2.94	2.44	2.92	3.02	3.06	3.08
		7	19	20	19	21	19	21	21	21	19	3.20	3.17	3.11	3.12	3.07	2.93	2.39	2.94	3.02	3.05
		8	19	20	20	20	19	19	21	22	20	3.18	3.14	3.15	3.13	3.12	3.07	2.95	2.35	2.92	2.99
		9	19	21	20	19	20	19	21	21	20	3.17	3.19	3.18	3.12	3.11	3.06	3.07	2.90	2.35	2.85
		10	21	20	20	21	19	21	21	18	19	3.11	3.11	3.12	3.12	3.11	3.11	3.06	3.00	2.89	2.37

20	380	1	21	19	21	21	20	20	20	17	19	20	2.48	3.00	3.05	3.11	3.08	3.09	3.04	3.04	3.06	3.06
			19	21	20	20	20	21	20	20	21		2.66	3.02	3.07	3.04	3.02	3.03	3.03	3.09	3.03	3.00
		2	20	20	21	19	21	20	20	20	20	20	2.98	2.61	2.99	3.04	3.04	3.04	3.04	3.04	3.04	3.06
			20	22	19	20	20	19	20	20	19		3.02	2.93	3.02	3.01	3.03	3.03	3.02	3.02	2.99	3.05
		3	21	21	21	21	21	20	19	20	19	21	3.09	2.93	2.38	2.97	3.04	3.06	3.06	3.05	3.05	3.01
			19	19	19	21	20	20	19	20	19		3.03	3.00	3.04	3.06	3.07	3.04	3.06	3.04	3.01	3.01
		4	20	20	20	22	20	19	21	20	21	19	3.04	3.06	2.99	2.64	2.96	3.04	3.02	3.00	3.04	3.02
			19	19	20	20	22	21	20	18	19		3.02	3.00	3.03	3.02	3.02	2.99	3.03	3.03	3.02	3.03
		5	21	20	20	19	22	20	19	20	21	19	3.05	3.08	3.04	3.01	2.65	2.96	3.02	3.03	3.04	3.03
			21	20	19	19	20	20	20	21	19		3.02	3.02	3.02	3.02	2.97	3.02	3.01	3.01	3.01	2.99
		6	20	20	20	20	21	21	20	19	21	20	3.02	3.02	3.03	3.04	2.92	2.68	3.00	3.03	3.03	3.01
			20	19	20	20	20	19	20	19	21		3.01	3.02	3.01	3.04	3.02	3.02	3.01	3.03	3.04	3.02
		7	21	19	21	19	23	21	20	21	21	19	3.01	3.01	3.05	3.08	3.00	3.00	2.66	2.99	3.06	3.02
			19	19	18	19	20	19	21	20	20		3.02	3.04	3.02	3.01	3.01	3.02	3.02	2.98	3.00	3.00
		8	20	21	20	20	19	20	20	23	20	20	3.01	3.08	3.07	3.06	3.09	2.99	3.02	2.74	2.96	2.97
			21	19	20	20	20	18	20	19	20		3.02	3.04	3.03	3.00	3.00	2.96	2.97	3.01	2.99	2.99
		9	23	19	19	20	19	19	23	16	22	20	3.07	3.03	3.05	3.06	3.07	3.04	3.06	2.96	2.69	2.94
			21	20	20	20	20	20	21	19	19		2.98	3.02	3.03	3.03	2.99	2.99	3.05	2.97	2.99	2.98
		10	19	20	21	20	20	19	20	20	19	23	3.06	3.09	3.08	3.09	3.07	3.07	3.05	3.05	3.01	2.41
			19	21	21	20	19	21	20	19	19		2.93	3.02	3.02	2.97	3.00	2.98	3.02	3.04	3.03	3.01
		11	20	20	21	21	19	19	19	18	21	22	3.01	3.09	3.04	3.06	3.06	3.07	3.09	3.07	3.11	2.91
			21	19	20	19	20	22	19	20	20		2.40	2.93	2.95	3.02	3.04	3.05	3.04	3.00	3.04	3.02
		12	21	20	20	20	21	21	20	19	20	21	3.11	3.07	3.07	3.10	3.10	3.06	3.06	3.07	3.08	3.01
			21	23	20	18	19	20	19	18	19		2.91	2.39	2.88	3.01	3.02	3.01	3.03	3.02	2.99	3.01
		13	22	20	19	20	21	20	20	20	20	19	3.08	3.06	3.09	3.10	3.10	3.07	3.08	3.03	3.07	3.10
			19	22	22	18	19	19	19	19	22		3.02	2.88	2.26	2.92	3.00	3.03	3.03	3.00	3.03	3.05
		14	20	22	22	19	20	21	20	20	19	20	3.08	3.07	3.07	3.10	3.07	3.11	3.11	3.11	3.10	3.03
			20	19	21	22	21	20	20	18	19		3.01	3.00	2.88	2.35	2.89	2.97	3.01	3.01	3.02	3.01
		15	20	21	21	21	20	20	22	20	21	18	3.04	3.10	3.06	3.09	3.11	3.06	3.11	3.06	3.08	3.09
			19	20	18	20	20	20	19	21	19		3.07	3.11	2.97	2.83	2.34	2.81	3.02	3.04	3.01	3.00
		16	20	19	21	20	21	19	20	21	20	21	3.08	3.05	3.13	3.08	3.09	3.08	3.10	3.07	3.08	3.13
			19	18	21	20	20	21	19	21	19		3.01	3.08	3.05	3.00	2.87	2.31	2.85	2.95	3.00	2.99
		17	19	22	19	19	22	21	21	19	20	18	3.06	3.12	3.10	3.08	3.09	3.11	3.06	3.08	3.07	3.10
			21	20	20	20	18	20	23	19	19		3.07	3.09	3.02	3.03	2.98	2.86	2.31	2.84	2.95	2.98
		18	19	19	18	20	21	21	20	21	21	20	3.10	3.10	3.07	3.08	3.10	3.11	3.06	3.12	3.11	3.08
			19	19	19	19	21	21	20	22	20		3.07	3.09	3.01	3.06	3.04	2.97	2.76	2.28	2.85	2.94
		19	20	20	21	20	19	20	20	21	18	19	3.09	3.08	3.07	3.08	3.09	3.09	3.07	3.07	3.08	3.11
			20	20	21	20	21	20	20	20	20		3.09	3.09	3.07	3.04	3.01	3.00	2.95	2.82	2.30	2.80
		20	19	20	20	19	19	22	21	20	19	21	3.13	3.10	3.10	3.06	3.08	3.08	3.03	3.07	3.07	3.07
			19	19	21	21	22	18	20	19	21		3.08	3.06	3.02	3.02	3.02	3.03	3.00	2.91	2.80	2.27

Note: Grey font in the cells is to highlight some values as indicated at the beginning of sub-sections 5.4.1 and 5.4.2. The row with a green font is used to highlight an example (case 6) as indicated near the end of subsection 5.4.1.

#### 5.4.1 Impact of having a machine with a different repair time

Table 5 presents the simultaneous buffer and service time allocations for a system where the failure times of all machines are identical and follow a geometric distribution with MTBF = 70, and where the repair times of the machines are different. For each case k, only machine *k* has a different repair time (MTTR = 30) compared to the other machines (MTTR = 10). The values of service time of the machine with a smaller MTTR and the corresponding downstream stock are marked in a grey font.

The results show that the machine which has a larger repair time needs less service time than the other machines and therefore, the downstream buffer size is larger (see the grey cells). These pattern allocations are more pronounced for a production line composed of three machines. For example, in case 1 (resp. case 3), the value of the first stock for the obtained configuration reached is 28 (resp. 15), whereas the value of the second stock is 12 (resp. 25). We notice that when the middle machine has a larger MTTR, the values of the two stocks are roughly similar (19 and 21 when n = 3). We can then observe that when *N* increases, the stock allocation becomes more uniform but the downstream buffer size of the machine with a larger MTTR remains in most cases slightly larger than or equal to the other buffers. In all cases, the service time of the machine with a larger MTTR is significantly smaller compared to the other machines. It can also be noted that the upstream machine and the downstream machine have a reduced service time (forming a bowl-shaped configuration around these three machines). For example, in the highlighted case 6 for n = 10, the best allocation reached for the service times is (3.15, 3.20, 3.10, 3.09, **2.94, 2.44, 2.92**, 3.02, 3.06, 3.08) and the best allocation proposed for the buffer capacities is (20, 21, 21, 20, 20, **21**, 19, 18, 20). We note also that the upstream machine and the downstream machine have lower service times (2.94 and 292, respectively) compared to the other machines. This certainly helps to improve the fluidity of the system and to reduce the total production time, and consequently to avoid possible blockages.

#### 5.4.2. Impact of having a machine with a different failure time

Table 6 presents the simultaneous buffer and service time allocations for a system where all the machines have the same repair times (MTTR = 10), and for each case k, only machine k has a different failure time (MTBF = 100) compared to the other machines (MTBF = 70). The service-time values of the machine with a bigger MTBF and the downstream stock are marked in grey font. The following observations can be made.

- In all cases, the service time of the machine with the bigger MTBF is larger than the other machines.
- The service times of the downstream machines following the machine with the bigger MTBF tend to decrease towards the end of the line, and as the number of machines increases, a roughly uniform allocation occurs.
- In most cases, buffers slightly tend to accumulate towards the downstream buffer of the machine with a high MTBF, and as the number of machines increases, the buffer allocation follows a uniform allocation.

**Table 6.** Results if one machine has a different failure time.

N	B	Case	$b^{*}$									$t^{*}$									
3	40	1	20					20				3.13			2.91			2.96			
		2	22					18				2.98			3.09			2.93			
		3	20					20				2.96			2.86			3.18			
10	180	1	21	21	19	21	20	19	20	19	20	3.11	3.03	2.97	2.97	2.97	3.00	3.01	3.00	2.97	2.97
		2	19	20	19	24	20	20	19	18	21	3.06	3.15	3.02	2.99	2.98	2.97	2.98	2.94	2.96	2.95
		3	21	20	20	20	22	20	20	18	19	3.06	3.03	3.13	2.98	3.00	2.97	2.98	2.98	2.92	2.95
		4	19	19	19	21	20	20	20	22	20	3.03	3.02	3.03	3.09	3.01	3.00	2.96	2.95	2.96	2.95
		5	21	19	23	18	20	20	20	19	20	3.01	3.03	2.97	2.97	3.08	2.96	2.99	3.05	2.97	2.97
		6	21	21	22	21	18	19	20	19	19	3.03	3.02	2.98	2.98	3.02	3.09	2.97	2.97	2.97	2.98
		7	21	19	21	20	20	19	21	19	20	3.04	3.05	3.03	2.98	2.97	2.99	3.10	2.94	2.94	2.96
		8	20	19	20	21	21	20	20	20	19	3.06	3.01	2.99	2.95	3.00	2.94	2.96	3.12	2.99	2.98
		9	20	21	20	21	20	20	20	19	19	3.06	3.03	2.99	2.98	2.97	2.97	2.95	2.97	3.09	2.99
		10	20	20	21	20	19	21	20	19	20	3.04	3.03	2.99	2.99	2.99	2.99	2.97	2.98	2.94	3.08

20	380	1	20	20	19	21	20	20	20	20	21	20	3.05	3.00	3.03	3.01	2.96	2.99	3.00	2.99	2.98	3.01
			19	20	21	19	20	20	20	21	19		2.96	2.99	3.00	3.02	2.97	2.98	3.01	3.01	3.04	3.00
		2	19	20	20	20	20	21	20	20	22	19	3.06	3.06	3.03	3.03	2.99	3.05	3.00	3.03	2.99	3.00
			19	21	19	20	20	20	19	21	20		2.94	2.97	3.02	3.00	2.9	2.99	2.99	2.98	3.00	2.97
		3	20	21	21	20	21	20	20	19	21	20	3.03	3.03	3.10	3.04	3.05	3.01	3.02	3.01	3.04	3.02
			20	21	20	20	19	20	19	19	19		3.02	2.97	3.00	3.02	2.88	2.92	2.87	2.99	2.96	3.02
		4	21	20	20	21	20	21	21	19	20	19	3.03	3.02	3.04	3.08	3.02	3.01	3.03	3.02	2.99	3.01
			21	20	20	19	19	20	20	19	20		2.98	2.99	3.02	3.00	2.98	3.01	2.97	2.94	2.95	2.91
		5	19	19	21	21	18	20	20	20	19	21	3.03	3	3.04	3.03	3.05	3.03	3.04	3.01	3.02	2.96
			21	20	19	20	19	21	22	20	20		3.02	2.98	3.01	2.96	2.98	2.97	2.98	2.97	2.95	2.97
		6	20	20	19	20	21	21	19	19	20	20	3.05	3.04	3.03	3.02	3.01	3.07	2.99	3.05	3.02	2.97
			21	20	20	20	21	19	21	19	20		2.95	3.05	3.05	3.04	2.96	2.98	2.98	2.84	2.99	2.91
		7	22	19	22	21	19	19	20	22	20	19	3.07	3.08	3.02	3.05	3.02	3.09	3.09	3.05	3.04	3.00
			19	20	20	20	21	20	19	19	19		3.02	2.98	3.01	2.97	3.01	2.99	2.88	2.78	2.94	2.91
		8	19	19	19	19	21	21	20	21	21	20	3.04	3.06	3.04	3.02	2.98	3.07	3.06	3.07	3.04	2.99
			21	23	19	19	20	22	18	19	19		2.97	2.96	2.93	3.03	2.89	2.97	3.00	2.98	2.94	2.96
		9	21	22	20	19	18	21	19	20	19	21	3.01	2.96	3.03	3.03	3.01	2.98	3.01	3.00	3.05	3.01
			20	19	21	21	20	19	19	22	19		3.00	2.99	3.03	2.99	2.97	3.03	2.98	2.97	2.94	3.01
		10	21	19	20	22	19	20	19	19	18	19	3.06	3.05	3.05	3.06	3.03	3.02	3.02	3.03	3.03	3.07
			20	23	21	21	18	20	20	20	21		3.03	2.91	3.01	2.95	2.99	2.96	2.96	2.91	2.96	2.9
		11	21	20	20	19	19	19	22	19	20	21	3.05	3.03	3.06	3.05	3.04	2.99	2.98	2.99	3.02	3.00
			21	19	21	20	19	21	20	19	20		3.07	3.03	3.00	2.99	2.96	2.96	2.94	2.96	2.94	2.94
		12	20	20	19	20	19	20	21	20	20	22	3.01	3.03	3.03	3.02	3.03	3.04	3.02	3.04	3.03	3.01
			20	20	20	20	21	21	19	19	19		3.01	3.06	3.05	3.00	3.04	2.99	2.96	2.91	2.94	2.78
		13	21	20	21	21	19	20	19	20	20	19	3.04	3.01	3.02	3.06	2.99	3.04	3.04	3.02	3.02	2.96
			20	21	20	20	19	20	19	20	21		3.00	3.05	3.09	2.92	3.03	3.03	2.95	2.86	2.98	2.89
		14	20	19	21	21	20	21	19	19	20	20	3.05	3.03	2.96	3.04	2.92	3.04	2.99	2.99	3.00	2.97
			20	19	20	20	21	20	20	19	21		2.99	2.97	2.99	3.06	3.05	3.01	2.95	2.99	2.99	3.01
		15	21	20	21	21	21	21	20	21	19	19	2.99	2.98	3.00	2.99	3.01	2.98	3.02	2.97	3.01	2.99
			18	20	19	18	20	20	20	19	22		3.01	3.00	2.94	2.97	3.04	3.03	2.97	3.04	3.03	3.03
		16	20	20	18	21	18	22	20	22	21	20	3.03	2.98	3.01	3.02	3.00	2.99	2.96	3.02	3.00	2.99
			20	20	21	20	18	20	19	20	20		3.00	3.00	3.04	2.99	3.00	3.04	3.02	2.97	2.99	2.95
		17	19	19	21	20	20	21	20	21	19	19	3.00	3.03	3.00	2.97	3.01	2.95	3.00	3.02	2.96	3.02
			21	19	21	20	19	20	21	20	20		2.97	3.00	3.02	3.02	2.97	3.00	3.04	3.01	3.01	3.00
		18	20	20	19	18	20	20	21	21	19	19	3.03	3.02	2.98	2.99	3.03	3.01	3.01	2.97	3.00	3.01
			19	21	21	20	20	19	21	20	22		3.02	3.01	2.98	2.96	3.01	3.02	2.94	3.03	3.02	2.96
		19	22	19	20	21	19	18	21	19	23	22	3.00	3.04	2.96	2.99	3.02	3.00	3.07	3.07	3.05	3.00
			19	18	21	22	19	19	17	21	20		3.00	2.99	2.99	2.99	2.95	2.91	2.99	2.98	3.09	2.91
		20	22	22	19	21	20	20	22	21	19	19	2.99	3.02	3.03	3.04	3.01	2.96	2.98	2.97	2.94	2.91
			20	21	19	19	20	18	18	21	19		2.98	2.99	3.02	2.98	3.05	2.96	3.06	3.01	3.03	3.07

Note: Grey font in the cells is to highlight some values as indicated at the beginning of sub-sections 5.4.1 and 5.4.2.

### 5.5. Results on very large instances

There are very few studies devoted to the case of very large production lines. They indicate that it takes very long computation times to obtain competitive results (typically tens of hours). In other words, the computing time increases considerably with the number n of machines. We consider production lines with n in 50, 75, 100 with five runs. To investigate the buffer allocation scheme with respect to a uniform allocation, $B_{\text{max}}$ is chosen to be a multiple of the number of buffers.

Table 7 presents the results of GA-FPA. $B_{\text{max}}$ is given in the second column. Column 3 presents both the best allocations of the buffer capacities $b^{*}$ and the service times $t^{*}$. Computing times are given in the last column (in seconds).

**Table 7.** Results for very large production lines (involving 50, 75, and 100 machines).

N	$B_{\text{max}}$	$b^{*}$	CPU (s)
50	245	4 5 6 5 4 4 5 4 6 4 4 5 6 4 5 4 5 5 7 4 5 6 5 5 4	2360
		7 6 5 6 5 6 6 5 4 5 4 5 5 4 7 4 5 6 6 4 4 4 4 7	
	490	10 10 11 11 9 11 10 9 10 9 9 9 8 11 10 13 12 9 11 9 11 12 7 11 9	1879
		11 11 9 10 7 13 11 7 7 8 10 10 9 9 12 12 7 12 10 11 11 11 11 10	
	$B_{\text{max}}$	$t^{*}$	
	245	2.999 3.000 3.000 3.000 2.993 2.996 2.998 3.002 3.002 3.002 3.002 3.001 3.000 2.997 2.998 3.000 2.998 3.001 2.999 2.998 2.998 3.000 3.001 3.001 3.002 3.000 3.001 2.999 2.998 3.002 3.001 3.002 2.999 3.000 3.001 2.999 3.002 3.001 3.000 2.998 3.004 3.000 3.001 3.003 3.001 2.999 2.994 3.002 3.002 3.003	
	490	3.005 3.001 3.000 3.000 2.996 3.002 3.000 2.998 2.999 3.000 2.996 2.999 3.005 2.995 2.999 2.999 2.998 3.003 2.997 3.005 3.000 2.997 2.998 2.998 2.998 2.996 3.000 2.997 3.003 2.998 2.997 2.999 3.005 3.003 3.004 3.003 2.998 3.002 2.996 3.003 3.003 2.999 3.004 2.998 3.004 2.999 3.002 2.999 3.001 2.999	
*N*	$B_{\text{max}}$	$b^{*}$	
75	370	5 4 6 6 5 5 3 5 5 7 6 4 5 6 5 4 6 5 5 3 6 6 6 5 5 6 4 4 6 6 5 4 5 4 3 4 5 5 5 6 5 6 5 5 4 6 5 5 6 4 5 5 3 5 5 5 5 7 4 7 5 5 6 5 5 4 4	4406
		5 5 4 5 7 3 5	
	740	9 8 9 11 10 11 11 9 11 10 11 9 11 10 10 10 10 7 10 13 9 8 10 11 10 8 12 9 14 9 7 11 11 9 11 9 10 11 10 13 10 11 10 11 11 10 9 9 11 11 10 10 8 10 8 10 10	7205
		9 11 12 11 10 12 10 10 9 10 9 10 9 8 10 10 9	
	$B_{max}$	$t^{*}$	
	370	3.006 2.999 3.000 3.003 2.999 3.002 3.002 2.998 2.998 2.996 2.996 3.001 2.997 2.999 3.001 3.004 2.997 2.998 3.004 3.001 2.999 2.998 2.999 3.001 2.999 3.002 2.998 3.003 2.997 2.998 2.999 2.999 2.998 2.998 3.002 2.996 3.005 3.003 2.999 3.002 2.999 2.999 3.002 2.998 2.997 3.003 2.998 3.002 3.003 3.000 3.001 3.001 2.999 3.001 2.999 2.999 3.002 3.003 3.002 3.002 3.002 3.001 3.004 3.000	
		2.998 2.995 2.999 3.003 2.999 2.998 2.997 2.999 2.997 3.002 3.000	
	740	3.000 3.003 3.004 2.999 3.001 3.000 3.002 2.999 2.998 2.999 2.997 2.998 3.000 2.999 2.999 3.003 3.001 2.999 3.000 2.998 3.000 2.998 3.000 2.998 3.001 3.001 3.000 2.998 2.999 3.000 3.000 3.001 2.998 3.000 3.002 3.004 2.997 3.000 2.999 3.000 3.004 3.001 2.999 2.997 2.998 3.000 3.000 2.998 2.999 3.003 3.004 3.003 3.002 2.999 3.000 3.006 3.000 2.999 2.997 2.998 3.002 3.003 3.000 3.000	
		3.000 2.998 2.997 3.000 3.000 3.002 2.998 2.999 2.998 3.001 3.000	
*N*	$B_{\text{max}}$	$b^{*}$	
100	495	5 5 4 5 4 6 4 6 5 5 5 6 4 4 6 4 5 4 4 5 6 4 5 5 5 5 4 5 6 5 6 8 5 4 6 5 4 5 5 4 4 4 6 4 6 5 4 5 5 6 5 5 4 5 5 5 4 4 6 6 7 4 5 5 5 4 4 6 4 5 5 5 5 7 4 5 4 7 7 4 6 5 4 5 7 6 6 5 5 6 4 5 5 5 4 5 5 6 3	10,029
	990	10 10 10 9 8 10 11 10 10 7 11 9 10 11 9 12 12 10 11 8 11 9 12 11 9 9 11 8 9 9 11 9 8 9 9 10 11 8 11 10 10 11 10 10 11 9 11 9 9 10 9 10 11 9 11 11 11 12 12 10 8 10 13 10 10 13 11 9 9 10 9 10 11 11 11 10 11 9 11 11 10 10 9 11 9	12,944
		11 10 11 7 10 8 9 9 12 8 11 8 11 11	
	$B_{\text{max}}$	$t^{*}$	
	495	3.003 2.997 2.999 3.002 3.001 2.999 3.002 3.000 3.002 3.001 3.002 2.999 2.999 2.995 2.998 2.999 2.995 3.003 2.999 3.000 3.000 3.001 3.000 3.000 2.999 3.002 2.998 2.998 2.999 3.000 3.002 3.000 3.000 2.998 3.002 2.999 2.999 3.000 3.000 2.999 3.001 2.998 3.001 2.997 3.001 3.001 3.003 2.998 2.997 3.002 2.999 3.003 3.001 2.997 3.002 3.002 2.999 3.001 3.004 3.003 3.000 2.999 3.001 2.998 3.000 3.000 2.999 3.002 3.001 3.002 2.997 3.001 2.997 3.002 3.001 2.999 3.002 3.001 3.000 2.999 3.000 3.002 2.996 3.001 3.000 2.999 3.001 3.002 3.000 3.000 3.002 3.000 2.999 3.000 3.002 2.998	
		2.999 3.002 2.999 2.996	
	990	3.000 3.000 3.000 3.000 3.000 3.000 3.000 3.000 3.002 3.000 3.001 3.001 3.000 3.000 3.000 3.000 2.999 2.999 3.001 3.000 2.996 3.002 2.998 3.002 3.002 3.001 3.001 3.000 3.000 2.998 3.000 3.003 3.001 3.000 3.001 3.000 3.000 2.997 3.000 2.999 3.003 3.001 3.001 3.001 3.000 2.999 3.001 2.998 3.002 3.002 3.001 3.000 3.000 3.000 3.000 3.001 2.999 3.001 2.999 2.997 2.999 3.001 3.000 2.997 2.999 2.997 3.002 3.000 3.001 3.002 2.997 2.998 3.000 2.998 2.997 2.998 2.999 3.001 3.000 2.995 3.002 3.001 3.001 3.000 2.998 3.000 3.000 3.000 3.001 3.001 3.000 3.000 3.000 3.004 2.999 3.001	
		3.001 3.001 2.998 3.000	

From Table 7, we can observe that the buffer allocation (column 3) does not follow the inverted bowl pattern, but the buffer capacities are almost all equal. Thus, it appears that the buffer values for most available physical locations are equal to the average value (i.e. $\theta_{i}=5$ or $\theta_{i}=10$, for i=1,…,n−1, where n is the number of machines) with a variation of ±2 units. This is certainly due to the nature of failure of the machines because if the system is symmetrical (by its structure and its operating parameters), the optimal allocation result should also be symmetrical (Cheikhrouhou, Paris, and Pierreval 2002).

Regarding the allocation of service times, the same observation can also be noticed. Indeed, the patterns of service times resulting from the optimisation do not follow the bowl-shaped allocation as in the case of small lines, but the service times of all machines are almost equal. Unlike the balanced case, where the characteristics of all machines are identical and the optimal allocation should be occurring when all service times $\theta_{i}$ ( i=1,…, n) are the same ( $\theta_{i}=3$ units in our case), the results of $t^{*}$ (column 3 of Table 7) present some slight deviation from this value (i.e. $\theta_{i}=3$ units). This can be explained by the fact that the machines are prone to breakdowns. We can also notice that this deviation is much lower (about $10^{-3}$) for the very large production lines (i.e. n = 50, 75 and 100 machines) compared to the large production lines (i.e. n = 40 machines), for which it is of the order of $10^{-2}$.

In summary, the patterns for buffer capacities and service times for very large production lines seem to follow roughly a uniform allocation. These results are in line with results of Spinellis, Papadopoulos, and Smith (2000) and can be explained by the symmetrical structure of the production line.

## 6. Conclusion

This study presents a robust hybrid technique coupling Finite Perturbation Analysis (FPA) with Genetic Algorithm (GA) for allocating simultaneously service times and buffer capacities in production lines with unreliable machines. The processing (service) time of each machine and the capacity of each buffer (between machines) must be determined while satisfying the total buffer-space and service-time constraints. The goal consists in maximising the production rate of the production line. FPA (based on the estimation of the production rate's gradients) forms the core of the proposed approach, whereas GA provides its input solutions. Thanks to the exploration and diversification ability of GA, the solution-space regions of the (near-)optimal solutions can be quickly reached. Next, benefiting from the exploitation and intensification ability of FPA, these solution-space regions are deeply investigated to find better solutions. Another benefit of the proposed method relies in being able to use a single simulation for the optimisation. Moreover, our experiments for instances with up to 100 machines show that the buffer and service-time allocation patterns (over the machines) corroborate previous patterns found in the literature but also exhibit some striking differences. These allocation patterns, while context dependent, are one of the most important insights for decision makers in designing production lines.

New solutions can be investigated (through the emergence of Industry 4.0 and the progress in manufacturing technologies) to improve the performance and the production rates of these systems. Indeed, the rise of intelligent and interconnected industrial robots in charge of executing system operations is helpful for monitoring inventory levels as well as tracking these different operations (including items/units transfer between operators/robots and the time needed to achieve it). In fact, the proposed approach, which allows for the simultaneous allocation of operator/robot service times and buffer capacities, offers new and interesting perspectives to enhance the performance of such systems. In order to have an in-depth and comprehensive analysis of unreliable production lines, another possible direction for this research is to use different criteria to account for different line combinations. Therefore, the proposed method might require important adjustments to extend it to other production systems such as transportation systems, assembly/disassembly systems or transfer lines.

## Data Availability

The paper includes no data.
